# Supplementary material for: Dynamic transcriptomic profiles of zebrafish gills in response to zinc supplementation
Source: BMC Genomics. 2010 Oct 11;11:553. doi: 10.1186/1471-2164-11-553 (PMC3091702; doi:10.1186/1471-2164-11-553)
Supplement: Additional file 2 — Interactive Direct Interaction Network representing the molecular interactions between zinc, copper, iron, calcium and proteins encoded by transcripts changed by zinc supplementation. Mini web-site containing index.html and hyperlinked pages in subdirectory describing a Direct Interaction Network automatically generated based on curated interactions contained within the proprietary PathwayArchitect database. Ovals represent proteins and the circles symbolize metal ions. Objects are coloured by their abundance in zebrafish at the time-point they were significantly different from the control is a scale from -4 fold (dark green) to +4 fold (dark red). Where significant differences were found at more than one time-point, the colour overlay shows expression at the first instance. Dark blue squares denote 'binding', and light blue squares 'expression'; green squares stand for 'regulation', green diamonds for 'metabolism', and green circles for 'promoter binding'. Arrow heads indicate directionality of the interaction where annotated. All nodes and edges can be further interrogated by selecting the relative area of the image. [file 1471-2164-11-553-S2.zip › PathwayArchitect Zn xs DIN/411300.html]

# BINDING:

|  |  |
| --- | --- |
| Type | BINDING |
| Effect | None |


---

|  |  |
| --- | --- |
| Score | 0 |


---

|  |  |
| --- | --- |
| Reference Count | 9 |


---

|  |  |
| --- | --- |
| Mechanism | Unknown |


---

|  |  |
| --- | --- |
| Reference:0 || Sentence | "Camostat treatment markedly increased the phosphorylation of both PHAS-I and eIF4E and the formation of eIF4E-eIF4G complex." |
| PMID | 11113094 |
| Year | 2000 |
| Species | Rat |
| Journal | Gastroenterology |
| RefScore | 1 |
| Source | PArchNLP |
  |
|  |  |


---

|  |  |
| --- | --- |
 Reference:1 || Sentence | "Refeeding a complete diet also stimulated the binding of eIF4E to eIF4G to form the active eIF4F complex." |
| PMID | 9398653 |
| Year | 1997 |
| Species | Rat |
| Journal | Biochem Biophys Res Commun |
| RefScore | 1 |
| Source | PArchNLP |
  ||  |  |


---

|  |  |
| --- | --- |
 Reference:2 || Sentence | "In muscle, protein synthesis is regulated through modulation of the binding of eIF4E to eIF4G and in liver through modulation of both phosphorylation of eIF4E as well as binding of eIF4E to eIF4G." |
| PMID | 9398653 |
| Year | 1997 |
| Species | Rat |
| Journal | Biochem Biophys Res Commun |
| RefScore | 1 |
| Source | PArchNLP |
  ||  |  |


---

|  |  |
| --- | --- |
 Reference:3 || Sentence | "In part, the increased binding of eIF-4G to eIF-4E was a result of release of eIF-4E bound to the translational regulator, PHAS-I, through a mechanism involving enhanced phosphorylation of PHAS-I." |
| PMID | 9124320 |
| Year | 1997 |
| Species | Rat |
| Journal | Am J Physiol |
| RefScore | 1 |
| Source | PArchNLP |
  ||  |  |


---

|  |  |
| --- | --- |
 Reference:4 || Sentence | "Overall, the results suggest that insulin stimulates protein synthesis in gastrocnemius muscle through a mechanism involving increased binding of eIF-4G to eIF-4E, which is in part due to phosphorylation of PHAS-I, resulting in a release of eIF-4E from the inactive PHAS-I x eIF-4E complex." |
| PMID | 9124320 |
| Year | 1997 |
| Species | Rat |
| Journal | Am J Physiol |
| RefScore | 1 |
| Source | PArchNLP |
  ||  |  |


---

|  |  |
| --- | --- |
 Reference:5 || Sentence | "This was correlated with an increase in the formation of the eIF4E-eIF4G complex after refeeding." |
| PMID | 15117679 |
| Year | 2004 |
| Species | Mouse |
| Journal | Am J Physiol Gastrointest Liver Physiol |
| RefScore | 1 |
| Source | PArchNLP |
  ||  |  |


---

|  |  |
| --- | --- |
 Reference:6 || Sentence | "Somatostatin attenuated the leucine-induced changes in 4E-BP1 and S6K1 phosphorylation and completely blocked the change in rp S6 phosphorylation but had no effect on eIF4G small middle dot eIF4E assembly." |
| PMID | 11934675 |
| Year | 2002 |
| Species | Rat |
| Journal | Am J Physiol Endocrinol Metab |
| RefScore | 0 |
| Source | PArchNLP |
  ||  |  |


---

|  |  |
| --- | --- |
 Reference:7 || Sentence | "A significant linear relationship existed between muscle IGF-I and the rate of protein synthesis or the amount of eIF4E bound to eIF4G." |
| PMID | 10827017 |
| Year | 2000 |
| Species | Rat |
| Journal | Am J Physiol Endocrinol Metab |
| RefScore | 0 |
| Source | PArchNLP |
  ||  |  |


---

|  |  |
| --- | --- |
 Reference:8 || Sentence | "Indinavir did not affect the phosphorylation of mTOR or 4E-BP1, but it did decrease the amount of the active eukaryotic initiation factor eIF4G-eIF4E complex." |
| PMID | 15229102 |
| Year | 2004 |
| Species | Mouse |
| Journal | Am J Physiol Cell Physiol |
| RefScore | 1 |
| Source | PArchNLP |
  |


---

|  |  |
| --- | --- |
